# Optimal Experience and Optimal Identity: A Multinational Study of the Associations Between Flow and Social Identity

**DOI:** 10.3389/fpsyg.2016.00067

**Published:** 2016-02-19

**Authors:** Yanhui Mao, Scott Roberts, Stefano Pagliaro, Mihaly Csikszentmihalyi, Marino Bonaiuto

**Affiliations:** ^1^Department of Psychology of Developmental and Socialization Process, Sapienza University of RomeRome, Italy; ^2^Department of Psychology, Claremont Graduate University, ClaremontCA, USA; ^3^Department of Psychology, Seconda Università degli Studi di NapoliCaserta, Italy

**Keywords:** eudaimonistic identity theory, flow experience, optimal experience, self-defining activities, social identity

## Abstract

Eudaimonistic identity theory posits a link between activity and identity, where a self-defining activity promotes the strength of a person’s identity. An activity engaged in with high enjoyment, full involvement, and high concentration can facilitate the subjective experience of flow. In the present paper, we hypothesized in accordance with the theory of psychological selection that beyond the promotion of individual development and complexity at the personal level, the relationship between flow and identity at the social level is also positive through participation in self-defining activities. Three different samples (i.e., American, Chinese, and Spanish) filled in measures for flow and social identity, with reference to four previously self-reported activities, characterized by four different combinations of skills (low vs. high) and challenges (low vs. high). Findings indicated that flow was positively associated with social identity across each of the above samples, regardless of participants’ gender and age. The results have implications for increasing social identity via participation in self-defining group activities that could facilitate flow.

## Introduction

### Research Background

The relation of activities to identity has been stressed in the prior literature, arguing that the activities a person is engaged in are important for his or her own identity definition or strength (Waterman, 1992, 1993a,b). Since the empirical evidence is not systematic and, to our knowledge, is focused only on personal identity (e.g., Waterman et al., 2003; Coatsworth et al., 2005; Waterman, 2005; Schwartz, 2006; Sharp et al., 2007; Tietze, 2008; Mao et al., 2016), the present contribution seeks to bring more substantial and systematic quantitative evidence to bear regarding this relationship, as well as to test such a link with reference to the individual’s identity at the social level. The primary, general hypothesis is based on Eudaimonistic Identity Theory (e.g., Waterman, 1984, 1990a, 1992, 1993a, 2004); that is, the idea that an individual starts to recognize elements of his/her true self, including goals, values, interests, talents, and abilities, through participating in personally salient identity-related activities, which are also termed self-defining activities (e.g., Waterman, 2004; Coatsworth et al., 2005, 2006; Sharp et al., 2007). More specifically, among the different salient features of a person’s self-defining activity experience, feelings of personal expressiveness (Waterman, 1990a) and flow (Csikszentmihalyi, 1975, 1982, 1990) could be particularly relevant in this context. These two subjective experiences of both personal expressiveness and flow appear to be conceptually linked from the eudaimonistic perspective (Waterman, 1993a), and the empirical evidence on the degrees of correlation within both constructs can be traced (Waterman et al., 2003). However, the direct test of the relation of flow to personal identity is sparse, though there is an exception by Mao et al. (2016). On these bases, we hypothesized that the relationship between flow experience and the definition or strength of a person’s identity at the social level – through the participation in self-defining, group activities – would be positive. Specifically, in a study carried out on three different samples from various national backgrounds, we tested the hypothesis that the higher the degree of flow experienced during self-defining activities by an individual, the higher the perception or strength of his or her social identity. The study was guided by theories listed below.

### Theoretical Foundations

#### Eudaimonia and Eudaimonistic Identity Theory

Eudaimonia is a word of Greek origin that can be traced back to Aristotle’s (IV century B.C.) *Nicomachean Ethics*. It is comprised of the words “*eu*” (“*good*”) and “*daimōn*” (“*spirit*”) being interpreted in different ways as “*virtue*” or “*excellence*”, and “*phronesis*”. The word Eudaimonia has been commonly translated as happiness (Waterman, 1990b), and has for instance been used in reference to: the highest human good achievable by human action (Ryff and Singer, 2008); the various realization of one’s potential (Irwin, 1985); an intrinsically good state or activity constitutive of the good life (Fraser, 2014); and seeking to use and develop the best in oneself (Huta, 2011). Based on Aristotle’s *Nicomachean Ethics*, Norton (1976) remarks in his book *personal destines* that eudaimonism as an ethical theory calls individuals to recognize and live with their daimon or “true self”. According to Aristotle (1925) eudaimonia requires activity, so that it is not sufficient for a person to possess an unpotentiated ability or disposition. Aristotle’s philosophy on eudaimonia has provided philosophically grounding influence upon psychological researchers across the study of different constructs regarding an individual’s growth and fulfillment, for instance, *Personal Expressiveness* and *Intrinsic Motivation* (Waterman, 1993b; Ashforth, 1997); *Self-determination* (Deci and Ryan, 1985; Ryan and Deci, 2000, 2001); as well as *Parenting* (Huta, 2011). Eudaimonistic identity theory (Waterman, 1990a, 1992) is grounded in both Aristotle’s philosophy of eudaimonia (Norton, 1976) and theories of personality (Erikson, 1968; Maslow, 1968; May, 1969). Erikson (1968, 1980) proposed that an optimally developed identity emerges from the discovery of the critical elements of one’s self, resulting in a sense of feeling at home with who one is and what one is doing in life. Individuals participate in a wide range of activities, some structured and some not so, and the eudaimonistic identity framework focuses on those self-defining activities (personal salient identity-related activities) that have the greatest likelihood of influencing identity, based on the fact that a person begins to discover and recognize his/her daimon or true self by means of flourishing through his/her interests, talents and abilities (Waterman, 1984, 1990a, 1992, 1993a, 2004; Coatsworth et al., 2005; Sharp et al., 2007). Thus, the eudaimonistic identity perspective provides a link for understanding the connection between identity experiences and a range of self-defining activities.

#### Self-Defining Activities

Activities that are important for identity development are those that provide the impetus toward self-actualization, which involves a developmental process tending toward the actualized version of one’s self, specifically, a “full use and exploration of talents, capacities, and potentials” (Maslow, 1970, p. 50). According to Erikson (1968, 1980), an optimal identity emerges from discovering the critical elements of one’s self that result in the understanding of who an individual is and what he/she is doing in life. Waterman (2004) suggests that it is through a process of exploration within identity-related activities that a person forms the basis for building a coherent personal identity. Such identity-related activities are identified by an individual as being important to who he/she is and what he/she is like as a person, and are essential for identity consolidation within adolescence and emerging adulthood in providing a broad social identity (e.g., activities of jocks vs. brains; Eccles and Barber, 1999) that suggests appropriate behaviors and how one fits into the social milieu (Coatsworth et al., 2005; Sharp et al., 2007). Recent research on identity development provides evidence that self-defining activities may assist adults in exploration of their sense of meaningful and coherent identity, facilitating the identity work necessary for identity integration, and providing a unique context for exploring their interests and talents (Barber et al., 2001; Fredricks et al., 2002; Hansen et al., 2003; Sharp et al., 2007). This literature has expanded the sense in which identity development is associated with action and is further informative for understanding the features of activities that promote identity strength, such as the experience of “flow”.

#### Flow, the Psychology of Optimal Experience

Seligman (2002) indicates that the good life, in alignment with Aristotle’s ‘eudaimonia’, is a life of engagement within different domains (i.e., family, work, love, and leisure). This state of engagement is often phenomenologically described as a complex and highly structured state of involvement, absorption and enjoyment (Delle Fave et al., 2011a). The term “flow” conceptualized by Csikszentmihalyi (1975) describes the optimal experiences that are the most enjoyable in human life when engaging an activity with full involvement (Csikszentmihalyi, 1977, 1982, 1990). When performing an activity that provides challenges and requisite skills, and when both challenges and skills are high and in balance, an individual is not only enjoying the moment, but is also stretching his/her capabilities with the likelihood of learning new skills and increasing self-esteem and personal complexity. This process of optimal experience has been colloquially referred to as being “in the zone”, or “flow” (Csikszentmihalyi and Csikszentmihalyi, 1988; Csikszentmihalyi and LeFevre, 1989; Csikszentmihalyi, 1990), as it expresses the feeling of fluidity and continuity in concentration and action reported by most individuals. Flow happens only when performing an activity with clear goals, immediate and unambiguous feedback, and with an appropriate match between an individual’s skills and the situation’s challenge. When personal skills exceed the situation’s challenge, boredom is the consequence (based on the Quadrant Model of the flow state); if challenges overpass an individual’s skills, anxiety evolves. If both challenges and skills are relatively low, apathy will be experienced (Csikszentmihalyi and LeFevre, 1989). According to Csikszentmihalyi (1975, 1990, 1997), optimal experience may emerge in any situation in which there is an on-going activity, and is characterized by a merging of action and awareness, an expanded sense of time, a dropping out of self-consciousness, a feeling of being in control of his/her action and of the environment, a total concentration on the task at hand, and intrinsically rewarding action without external reward to maintain the behavior. Flow has been researched worldwide since the 1980s (e.g., Csikszentmihalyi and Csikszentmihalyi, 1988; Massimini and Carli, 1988; Csikszentmihalyi, 1990; Inghilleri, 1999; Massimini and Delle Fave, 2000). A large number of researchers have corroborated evidence for flow experience within the performance of a large number of different activities, such as, chess playing, rock climbing and dance (Csikszentmihalyi, 1975, 2000); sports (Jackson and Marsh, 1996; Kowal and Fortier, 1999; Harmison, 2006), music and the arts (Csikszentmihalyi and Csikszentmihalyi, 1988; Csikszentmihalyi and LeFevre, 1989); work (Csikszentmihalyi, 1988; Demerouti, 2006); and military and police combat (Nakamura and Csikszentmihalyi, 2002; Harari, 2008). Flow experience induced from these and other activities can have far-reaching implications in supporting individual growth, and is essential to the process of individuals’ identity formation, based on the fact that it increases intrinsic motivation, while organizing and activating thoughts and behaviors that contribute to the affirmation and construction of their self-definitions (Waterman, 2004). Further, when increasingly challenging activities are being enjoyed, the individual is more likely to develop his/her identity as having more control with increased capability (Csikszentmihalyi and Rochberg-Halton, 1981) when in flow. Age or gender effects, though they sometimes appear, are not consistent, and flow is expected to happen no matter the age or gender of the person, provided that the necessary conditions for that person are in place (Csikszentmihalyi, 1990).

#### Psychological and Bio-Cultural Selection

Optimal experience is not only an end in itself in being one of the most enjoyable of human experiences, yet also carries impact as being a producer of both the construction and complexification of the self, as well as a driving factor in the selection of bio-cultural information, which contributes to cultural evolution (Csikszentmihalyi and Csikszentmihalyi, 1988, 2006; Massimini and Delle Fave, 2000; Delle Fave et al., 2011a). The process of preferentially selecting attentional foci, which promote the cultivation of an individual’s talents and skills, is known as “psychological selection,” and this orientation leads to a trajectory of skill development and cultivation of the activities that provide platforms for personal growth (Csikszentmihalyi and Massimini, 1985). Because of the inherently enjoyable and beneficial qualities of flow experiences, the activities which promote optimal experience are more likely to be selected for engagement by the individual and are also more likely to become established within a cultural repertoire (Massimini and Delle Fave, 2000). From an individual’s perspective, the cultivation of optimal experiences is seen to be advantageous to personal development, and this evolutionary perspective has been turned upon the culture at large to examine how psychological selection applies at the level of society (Delle Fave et al., 2011b). When the relatively greater replication of optimal experience as compared to other experiences is examined from the social viewpoint, the bio-cultural theoretical assumptions that underlie this mechanism (Csikszentmihalyi and Massimini, 1985) extend the case that these adaptations are advantageous at a cultural level (Csikszentmihalyi and Csikszentmihalyi, 1988). A culture that provides the contexts necessary for individuals to engage in optimal experience, benefits by the complexity and growth which is fostered, and also benefits by the sharing of these experiences with others, and in both respects optimal experience could foster a greater sense of psychological and socio-cultural adjustment across a variety of cultures and domains (Delle Fave and Bassi, 2009; Delle Fave et al., 2011b). If the bio-cultural theoretical assumptions that undergird the application of psychological selection to the cultural domain hold true, then the social identification with a group beyond the individual self may provide the bridge between an individual and the relationships, groups, and cultures that foster optimal experience.

#### Social Identity Approach

The social identity approach (for a recent review, see Reicher et al., 2010) encompasses two related theories, that is, Social Identity Theory (Tajfel and Turner, 1979, 1986) and Self-Categorization Theory (Turner et al., 1987). The meta-theoretical approach of social identity points to the recognition that human beings are psychological group members, acting in terms of shared social identities – that is, the portion of an individual’s identity stemming from belongingness to different groups, together with the emotional reaction and the cognitive evaluation associated with this belongingness. Importantly, the social identity approach looks at the way in which a person behaves in a group as regulated by a distinct level of self, a higher-order self. This means that an individual within different social contexts does not lose his/her self, but simply shifts to a higher order, more socially inclusive representation of himself/herself as part of a relevant group. In order words, he/she acts in terms of his/her social identity, an aspect of identity that reflects a person’s sense of who he or she is based on his or her group memberships (e.g., within an organizational context, Ashforth and Mael, 1989). Thus, the social identity approach posits that, under certain circumstances, individuals think, feel, and act as members of collective groups, organizations, and cultures, and that an individual’s behavior reflects these larger societal units. For example, according to Turner (1982) a personal identity is comprised of an individual’s unique and personal concepts or characteristics, while a social identity involves membership in social groups or categories. Personal (e.g., the individual’s sense of self-concept) and social (e.g., the salience of group membership in the context) factors influence an individual’s focus on the different levels of his/her identity. The more that the individuals identify with a group, the more that they have a sense of belongingness with that group, and their attitude and behavior are then governed by their group membership (Ashforth and Mael, 1989). Group activities in which individuals in a society cognitively categorize themselves and others form the basis upon which the individuals obtain both their sense of self-esteem and interpersonal belongingness (Albert et al., 1998). Individuals are thusly driven to actively engage in interactions with others who share similar goals, values, interests, and beliefs. This process can be extended to larger social entities such as a person’s living and activity place, that is, the social and physical context for carrying out one’s own activities and being engaged in them (Twigger-Ross et al., 2003). Human beings are seen to be innately social animals interacting with each other by means of a vast assortment of differential activities, and social identities are developed by way of activity participation (Waterman, 1990a; Eccles and Barber, 1999; Fredricks et al., 2002; Hansen et al., 2003). Not all activities in which individuals participate provide the subjective experiences (e.g., flow) that lead to identity development. Self-defining activities, which participants identify as being important to who they are as a person, may be particularly essential for identity development, insofar as they provide opportunities to explore whether a specific social identity and all that goes with it is comfortable and consonant with one’s conceptions of their “true self” (Coatsworth et al., 2005).

### Evidence on the Association Between Flow and Identity

A few bodies of literature have addressed both constructs of flow and personal expressiveness, which to some extent could be considered as a proxy for personal identity. For instance, Waterman et al. (2003) measured the variables of interest, flow, and feelings of personal expressiveness, and found that these variables were strongly inter-related when predicting the subjective experience of intrinsic motivation, with the correlation between flow and personal expressiveness being 0.50 and 0.66, respectively in two sub-studies. Coatsworth et al. (2005) reported a correlation of.55 between flow and personal expressiveness when exploring adolescent self-defining leisure activities and identity experiences across three countries. A later investigation by Waterman (2005) remarked that the category of high-effort, high-enjoyment activities was associated with a greater reported level of both flow and personal expressiveness than those with low-effort and low-enjoyment activities, however, the links between flow and personal expressiveness had not been quantitatively tested. Schwartz (2006) indicated a correlation of.25 between self-discovery of identity (three levels: flow, personal expressiveness, interest) and ego identity when predicting identity consolidation from self-construction, eudaimonistic self-discovery, and agentic personality. The authors suggested that there might be a relation between flow and personal identity, though it had not yet been tested. A study by Sharp et al. (2007) tested gender and country differences for adolescent and emerging adults in examining subjective identity-related experience (e.g., flow, intrinsic motivation, and personal expressiveness) within self-defining activities. The results showed that identity-related experiences differ significantly across seven broad activity classes, and that personal expressiveness was greatly associated with self-defining activities when experiencing flow. However, the association between flow and personal identity was not clearly stated or operationalized. A qualitative case study by Tietze (2008) maintained that engagement in jazz music could facilitate not only flow but also a feeling of strong personal identity. The most recent study by Mao et al. (2016) has both quantitatively and explicitly tested the constructs of flow and personal identity, and found positive significant correlations (*r*s ranged from 0.43 to 0.84) between these two constructs within four different types of self-defining activities.

Although flow has been marked as a salient feature in experiencing self-defining activities that can thereby contribute to identity formation at the personal level, until now almost no literature has explicitly addressed the relationship between flow and identity at the social level. Further, previous literature linking personal identity with activities has chiefly focused upon participation in structured school-based extracurricular activities that were organized on behalf of the youth (Eccles et al., 2003; Hansen et al., 2003; Coatsworth et al., 2005). Identity development, however, is likely to occur in a wide range of activities, both structured and unstructured that individuals do alone or with other people, such as playing music, rock climbing, paid work and writing a paper (Csikszentmihalyi and Kleiber, 1991; Kleiber, 1999; Waterman, 1993b). Hence, the present study seeks to extend the previous literature on personal identity by exploring the associations between flow and social identity through a variety of self-defining activities.

### Study Aims and Hypotheses

The current work aimed to extend the work of Mao et al. (2016) regarding flow and a person’s identity on the individual level, to a social identity level. As indicated by Sharp et al. (2007), a multi-national design is important for an understanding of the generalizability of models of identity development. Accordingly, we carried out a multi-national study, in which we explored the association between individuals’ flow experience and perceived social identity, as a result of engagement in four different self-defining group activities (i.e., Activity 1, 2, 3, 4) based on four combinations of skills and challenges. In order to realize this objective, this study was oriented by the following hypotheses.

(1) We anticipated that an individual’s perceived flow or social identity would neither be influenced by participant’s gender nor by age, when one engages in his/her reported group self-defining activities.
(2) According to the above reasoning, we predicted a main effect of the differentially self-defining, group activities on both participants’ perceived flow and social identity. These self-defining, group activities derived from the combinations of participants’ perceived skills (low vs. high) and (low vs. high) challenges.
(3) Crucially for the present purpose, we anticipated flow to positively correlate with perceived social identity when engaging in self-defining group activities.

## Materials and Methods

### Materials

Standard and specific scales were utilized to measure both flow and social identity, for each of the four different activities elicited by combinations of different levels of skills (low vs. high) and challenges (low vs. high). The instrument delivered by means of a self-report questionnaire consisted of three sections (See **Supplementary Data Sheet 1**). The first section included two open questions asking for generation of group self-defining activities. The second section was comprised of fifteen multiple-choice questions regarding flow experience and social identity. Most items were positively worded to express the presence of flow experience and perceived social identity, except one negatively worded item that was reverse coded. Responses were made on a 5-point Likert-type scale with answers ranging from “*Not at all characteristic of me*” to “*Completely characteristic of me”*. Finally, the third section was comprised of ten questions assessing participants’ social-demographic backgrounds.

#### Tools and Categorization for Self-Defining Activities

By adapting a similar administration technique made by Waterman (1993b), two questions were developed to generate the personally defined group activities regarding which the subject subsequently had to fill in the flow and social identity tools: *(a) If you wanted another person to know about who you are and what you are like as a group member, what group activities you regularly engage of importance to you would you describe (minimum 5 activities maximum 10 activities)? (b) From the above activities, please choose 4 (four) activities in which you regularly engage that are characterized by different combinations of skills (low vs. high) and challenges (low vs. high), and write the chosen activities in each of the following boxes*. With these two questions, 4 out of the 5 to 10 personally defined group activities were selected and placed in a 2 × 2 table by the relative standing. Based on the Experience Fluctuation Model of the flow state (Massimini and Carli, 1988), the 2 (skills vs. challenges) × 2 (low vs. high) table where subjects entered the four selected activities was framed in the following order: Activity 1 (*low skills and low challenges*) - corresponding to the state of *Apathy*; Activity 2 (*high skills and low challenges*) – corresponding to *Relaxation*; Activity 3 (*low skills and high challenges*) – corresponding to *Anxiety*; Activity 4 (*high skills and high challenges*) – corresponding to *Flow*. Each subject was left free about which group(s) to refer to in answering the questionnaire: he/she implicitly decided to which group(s) make reference to by simply selecting each group self-defining activity. Then the subsequent tools (flow and social identity) were administered for Activity 1, 2, 3, 4, respectively.

#### Flow Scale

An eight-item flow scale was adopted. These items were phrased as completions of a common stem anchored by *not at all characteristic of me* to *completely characteristic of me*, with *moderately* in the middle: “When I engage in this activity ____”, and the item completions were the following: (1) I feel I have clear goals, (2) I feel self-conscious (reverse scored), (3) I feel in control, (4) I lose track of time, (5) I feel I know how well I am doing, (6) I have a high level of concentration, (7) I forget personal problems, and (8) I feel fully involved. The scale has been widely used by Waterman and colleagues (Waterman et al., 2003, 2008; Schwartz, 2006; Schwartz and Waterman, 2006), and its structure was tested with a reported alpha coefficient ranging from 0.80 to 0.83 in a multinational sample testing the associations between flow and personal identity (Mao et al., 2016). Alpha for the present study concerning the American, Chinese and Spanish sample is 0.77, 0.88, and 0.82, respectively.

#### Social Identity Measures

To measure the perceived social identity we used two different scales. The first was a social identification scale named organizational identification (OID), and comprised six items adapted from Mael (Unpublished doctoral dissertation) and Mael and Ashforth (1992). The items were: (1) When someone criticizes my group, it feels like a personal insult. (2) I am very interested in what others think about my group. (3) When I talk about my group, I usually say ‘we’ rather than ‘they’. (4) When someone praises my group, it feels like a personal compliment. (5) My group’s successes are my successes. (6) If a story in the media criticized my group, I would feel embarrassed. The Cronbach’s alpha values in previous studies were 0.81 in a sample of employed business and psychology students Mael (Unpublished doctoral dissertation), and 0.83 in a sample of managers from a variety of organizations and hierarchical levels (Ashforth, 1997). From 0.83 to 0.84, as well as from 0.87 to 0.89, in two samples of squad leaders by utilizing only the first five items of this scale (Mael, 1989). Alpha for the present study concerning the American, Chinese, and Spanish sample is 0.90, 0.89, and 0.89, respectively.

The second tool for testing group/social identity is a direct, graphically based measure of OID taken from Bergami and Bagozzi (2000, alpha = 0.71); this single item was developed to measure the individuals’ visual perception on the overlap between his or her own self-concept and the identity of a specific organization. The single-item task was: *Imagine that one of the circles at the left in each row represents your own self-definition or identity and the other circle at the right represents your group identity, please indicate which case best describes the level of overlap between your own and your group identity*. The answers for this question were provided by eight graphical scales ranging from *Far Apart* to *Completely Overlap* with *Moderately Overlap* in the middle.

### Methods

#### Participants

The study sample was comprised of 284 participants from three different countries: American (110), Chinese (70), and Spanish (104). Participants’ socio-demographic features were reported in **Table 1.**

**Table 1:** Socio-demographic features of the participants.

Country	Gender	Age		Educational degree (%)				Region (%)	
				
	Male (%)	*M*_age_ (age range)	*SD*	High school	Bachelor	Master	Ph.D.	Urban	Sub-urban
US (*n* = 110)	59	36.8 (18–72)	13.95	46.4	41.8	10	1.8	31.8	51.8
CN (*n* = 70)	49	29.4 (21–50)	5.60	5.7	25.7	35.7	32.9	78.6	20
Spain (*n* = 104)	56	34.8 (18–62)	9.30	5.7	34.7	22.1	1.9	69.2	26


#### Procedure

The three-part English-language questionnaire that was administered online through Qualtrics Survey software for the US subjects was translated and then back translated to the Chinese and Spanish-languages, and subsequently launched online in a Google Documents environment, respectively, for consenting Chinese and Spanish participants, respectively.

Prior to data collection, the required forms for ethical research conduct were prepared and then submitted to the Claremont Graduate University Institutional Review Board (IRB) Office (CGU IRB #2161), at which point the project was approved in the US by routine exemption due to proper survey design, anonymity, and lack of harm to participants. As for the Chinese and Spanish samples, ethical principles were followed as based upon the guidelines from international scientific communities in Psychology and thusly ethics approval including subjects’ informed consent for participation was also provided. Due to the online nature of the experiment, for each experiment informed consent was administered to subjects online. As with such online surveys, subjects were presented the text that included risks, choice to participate, ability to leave, and then endorsed the item to signal their acceptance prior to proceeding with the survey.

The online questionnaires were administered by sending the hyper-link of the survey web sites after a first round of pilot testing. Participants were recruited in large urban or suburban areas in each country: participants from US were recruited via Claremont Graduate University in Los Angeles, the Chinese participants were recruited at Chongqing University in Chongqing China, and the Spanish sample was recruited from Madrid in Spain^1^. Data gathering lasted for 3 months from September 2013, by means of contacting the voluntary respondents in order to gain compliance and participation in our survey; subjects’ answers were collected and coded for statistical analysis with the SPSS computer software program. By using such a cross-national sample of adults, the study is designed to broaden the extant literature on flow and identity development at the social level, through experiencing self-defining group activities.

#### Data Analytic Strategies

##### Preliminary analysis

To examine the gender differences on flow and social identity we performed *Independent Sample t-test*, while we relied on *Univariate Analyses of Variance* to examine the age and educational background differences on both flow and social identity.

*Two-way analysis of variance* (*ANOVA*) was applied to check that the combinations of skills and challenges tapped distinctly different levels of flow, for each subject and each activity, and within the 2-level (low, high) 2-factor (skills, challenges) structure.

The main hypothesis (H3) concerning the association between flow and social identity was tested by means of *Pearson Correlation Coefficients*, based on the activity level (with respect of each activity), as well as on the individual level.

## Results

### Preliminary Results^2^

#### Effect of Gender on Flow and Identity

As is shown in **Table 2** where mean values and standard deviations were disaggregated by gender, results yielded from the independent sample *t*-tests for gender differences on flow regarding three different samples did not indicate any significant differences (all *p*s > 0.05). Yet as regards with two different social identity measures, no effect (all *p*s > 0.05) was detected either from gender on OID scale developed by Mael and Ashforth (1992), or on Bergami and Bagozzi’s (2000) graphical measure. Thus, gender was neither related with flow nor with social identity, as predicted in H1.

**Table 2:** Gender differences in flow and social identity.

	Sample by country	Males	Females	*t*	*p*	Effect size *d*
Flow	U.S.	3.61 (0.53)	3.64 (0.60)	0.31	0.76 (n.s.)	0.053
	CN	3.18 (0.58)	2.97 (0.59)	1.52	0.13 (n.s.)	-0.359
	Spain	3.52 (0.33)	3.54 (0.40)	0.31	0.75 (n.s.)	0.055
OID	U.S.	3.36 (0.79)	3.27 (1.03)	0.47	0.64 (n.s.)	-0.098
	CN	3.17 (0.72)	3.07 (0.79)	0.59	0.56 (n.s.)	-0.117
	Spain	3.2 (0.54)	3.39 (0.57)	1.72	0.09 (n.s.)	0.342
Graphical	U.S.	5.13 (1.25)	4.82 (1.23)	1.28	0.20 (n.s.)	-0.25
	CN	5.04 (1.16)	4.72 (1.17)	1.16	0.25 (n.s.)	-0.275
	Spain	4.82 (0.97)	4.87 (0.98)	0.24	0.81 (n.s.)	0.051


*OID is the organizational Identification scale developed by Mael and Ashforth (1992), and Graphical is the Bergami and Bagozzi’s (2000) graphical measure.*

#### Effect of Age on Flow and Social Identity

**Table 3** presents mean values and standard deviation disaggregated by age classes. *Univariate Analyses of Variance* with age as independent variable did not show any significant effects (all *p*s > 0.05) on flow or social identity concerning the American sample (recoded as follows: 18–29; 30–39; 40–49; 50–59; 60 and more), Chinese sample (i.e., 21–29; 30–39; 40–49), as well as the Spanish sample (i.e., 18–29; 30–39; 40–49; 50–59; 60; and more). Thus, again in line with H1, even age was unrelated to flow and social identity, as hypothesized.

**Table 3:** Age differences on flow and social identity regarding the three different samples (US, CN, Spain).

Dependent variable	Sample by country	18–29	30–39	40–49	50–59	60 and more	*F*	*p*	$\eta_{p}^{2}$
Flow	US	3.32 (0.79)	3.26 (0.84)	3.01 (0.89)	3.32 (0.44)	3.20 (0.49)	0.43	0.79	0.016
	CN	3.12 (0.57)	2.93 (0.74)	2.93 (0.15)	–	–	0.66	0.52	0.019
	Spain	3.49 (0.29)	3.55 (0.38)	3.46 (0.33)	3.73 (0.53)	3.19 (0.21)	1.14	0.34	0.044
OID	US	3.30 (1.00)	3.25 (1.07)	3.29 (0.96)	3.33 (1.11)	3.35 (0.79)	0.03	0.99	0.001
	CN	3.15 (0.76)	3.05 (0.83)	2.91 (0.22)	–	–	0.26	0.77	0.008
	Spain	3.07 (0.46)	3.33 (0.59)	3.39 (0.32)	3.50 (0.94)	3.70 (0.53)	1.95	0.11	0.073
Graphical	US	3.26 (1.02)	2.91 (0.97)	2.95 (0.93)	2.81 (1.14)	3.25 (0.71)	0.94	0.44	0.035
	CN	4.94 (1.29)	4.82 (0.78)	4.31 (0.31)	–	–	0.55	0.58	0.016
	Spain	4.55 (0.87)	5.03 (1.02)	4.94 (0.75)	4.94 (1.41)	3.75 (0.39)	1.56	0.19	0.059


*OID is the organizational Identification scale developed by Mael and Ashforth (1992), and Graphical is the Bergami and Bagozzi’s (2000) graphical measure of social identity.*

### Manipulation Check for Flow and Social Identity: Comparisons of Flow and Social Identity Within Different Activities

A repeated measures 2 × 2 ANOVA was conducted to examine the effect of skills and challenges level on the subjective experience of flow. The results yielded a main effect of skills [*F*(1,283) = 117.91, *p* < 0.001, $\eta_{p}^{2}$ = 0.29] indicating that the mean flow score was significantly greater for high skills (*M* = 3.62, *SD* = 0.57 than for low skills (*M* = 3.29, *SD* = 0.64), and a main effect of challenges [*F*(1,283) = 220.31, *p* < 0.001, $\eta_{p}^{2}$ = 0.44) indicating that the mean flow score was significantly higher in the high challenges (*M* = 3.83, *SD* = 0.66) than in the low challenges (*M* = 3.07, *SD* = 0.74). These effect were qualified by the predicted interaction between skills and challenges (*F*(1,283) = 26.53, *p* < 0.001, $\eta_{p}^{2}$ = 0.09). A *post hoc* analysis conducted with the LSD test showed that flow in Activity 4 was significantly higher than in other three kinds of Activity (all *p*s < 0.001). Moreover, flow in Activities 3 and 2 was reliably higher than in Activity 1 (both *p*s < 0.001), and flow in Activity 3 was significantly higher than in Activity 2 (*p* < 0.001). These findings revealed that high-skills activities (i.e., Activity 2, Activity 4) are associated with greater flow experience, and high-challenges activities (i.e., Activity 3, Activity 4) are associated with greater reported flow, in comparison with low-skills activities (i.e., Activity 1, Activity 3), and low-challenges activities (i.e., Activity 1, Activity 2), respectively. Such a manipulation check has proved that the combination of the corresponding 2 × 2 framework worked for differentiating the subjective experience of flow. Alternative data analysis by Pearson correlation between flow and activity (*r* = 0.47, *p* < 0.001) also serves to verify that the differing combinations of characteristics (skills vs. challenges) are associated with different levels of flow.

Likewise, a repeated measures 2 × 2 ANOVA was also applied to compare the social identity levels (two different measures) within four different activities. Specifically, for OID scale: the main effect of skills yielded an *F* ratio of *F*(1,283) = 23.08, *p* < 0.001, $\eta_{p}^{2}$ = 0.08 indicating that the mean value was significantly greater for high skills (*M* = 3.34, *SD* = 0.80) than for low skills (*M* = 3.17, *SD* = 0.82). The main effect of challenges yielded an *F* ratio of *F*(1,283) = 117.52, *p* < 0.001, $\eta_{p}^{2}$ = 0.29 indicating that the mean value was significantly higher in the high challenges (*M* = 3.59, *SD* = 0.91) than in the low challenges (*M* = 2.91, *SD* = 0.94). The interaction effect was also significant [*F*(1,283) = 27.53, *p* < 0.001, $\eta_{p}^{2}$ = 0.09). Similar findings were obtained from the other social identity measure by Bergami and Bagozzi (2000): a two-way ANOVA yielded a main effect for the skills, *F*(1,283) = 13.01, *p* < 0.001, $\eta_{p}^{2}$ = 0.04, such that the mean value was significantly higher for high skills (*M* = 3.15, *SD* = 0.78) than for low skills (*M* = 2.98, *SD* = 0.85). The main effect of challenges was significant, *F*(1,283) = 183.43*, p* < 0.001, $\eta_{p}^{2}$ = 0.39, such that the mean value was significantly higher for high challenges (*M* = 3.54, *SD* = 0.88) than for low challenges (*M* = 2.59, *SD* = 0.97). The interaction effect was also significant [*F*(1,283) = 24.32, *p* < 0.001, $\eta_{p}^{2}$ = 0.08). For both social identity measures, the results of the *post hoc* LSD pairwise comparisons showed a growing and significant trend from Activity 1 (*Apathy*) to Activity 4 (*Flow*). The levels of social identity in the *Flow* activity were significantly higher than in all the other three Activities (*Apathy*, *Relaxation*, and *Anxiety*; all *p*s < 0.001). The levels of social identity were significantly higher in the *Anxiety* activity than in the *Relaxation* and *Apathy* (both *p*s < 0.001). Finally, the differences between social identity in the *Relaxation* and *Apathy* approached significance (*p* = 0.05).

Additionally for each activity (Activity 1 to Activity 4), the mean value for flow and social identity across the subjects was varied (see **Table 4**). The varying trend (*Flow* > *Anxiety* > *Relaxation* > *Apathy*) from the present sample indicates that flow and social identity are best achieved when both skills and challenges are at their peak and are balanced, i.e., when engaging in Activity 4. Importantly, the high-challenges, high-skills activity category (Activity 4) is associated with greater reported level of flow and social identity than the high-challenges, low-skills activity (Activity 3). Such a manipulation check indicates that the combinations of different levels of skills and challenges are effective in distinguishing four different activity types based on these measures.

**Table 4:** Means and standard deviations for flow and social identity by activity extracted from repeated measures ANOVA (*N* = 284).

Variable	Activity 1 (Low S and Low C-Apathy)	Activity 2 (High S and Low C-Relaxation)	Activity 3 (Low S and High C -Anxiety)	Activity 4 (High S and High C-Flow)	*F*	*p*
Flow	2.97 (0.84)	3.17 (0.79)	3.60 (0.73)	4.07 (0.74)	111.64	0.000
OID	2.89 (1.06)	2.93 (1.06)	3.40 (0.98)	3.78 (1.01)	48.54	0.000
Graphical	2.56 (1.09)	2.62 (1.18)	3.34 (1.03)	3.74 (1.03)	79.15	0.000


*S stands for Skills, C means Challenges, OID is the organizational Identification scale developed by Mael and Ashforth (1992), and Graphical is the Bergami and Bagozzi’s (2000) graphical measure.*

### Relations Between Flow and Social Identity

**Table 5** presents the correlation between the scales of flow and social identity (Mael and Ashforth, 1992): In line with our main hypothesis, correlations between these two scales were significant and positive within each activity and across the three national samples on the individual level. The results indicate that whenever the experience of flow within an activity is indicative of a relatively higher social identity, this appears for all types of self-defining activities that participants reported in the questionnaire.

**Table 5:** Correlations between flow and OID (Mael and Ashforth, 1992).

Activity type	Correlation	US	CN	Spain
Activity 1 (Apathy)	Flow_Activity1 OID _Activity1	0.61^∗∗^ (*N* = 110)	0.79^∗∗^ (*N* = 70)	0.42^∗∗^ (*N* = 104)
Activity 2 (Relaxation)	Flow_Activity2 OID _Activity2	0.26^∗∗^ (*N* = 110)	0.61^∗∗^ (*N* = 70)	0.55^∗∗^ (*N* = 104)
Activity 3 (Anxiety)	Flow_Activity3 OID _Activity3	0.48^∗∗^ (*N* = 110)	0.73^∗∗^ (*N* = 70)	0.11^∗^ (*N* = 104)
Activity 4 (Flow)	flow_Activity4 OID_Activity4	0.45^∗∗^ (*N* = 110)	0.87^∗∗^ (*N* = 70)	0.32^∗∗^ (*N* = 104)
Across activities	Flow_Across activities OID_Across activities	0.55^∗∗^ (*N* = 110)	0.47^∗∗^ (*N* = 70)	0.27^∗∗^ (*N* = 104)


*^∗^*p* < 0.05; ^∗∗^*p* < 0.01.*

**Table 6** shows correlation coefficients between the flow scale and the Bergami and Bagozzi’s (2000) social identity scale, and the observed significant positive correlations between these two scales verify our hypothesis in H3. Such results again strongly revealed that whenever the experience of flow is greater during the activity, an individual’s perceived social identity is seen greater accordingly.

**Table 6:** Correlations between flow and Bergami and Bagozzi, 2000 graphical measure of social identity.

Activity type	Correlation	US	CN	Spain
Activity 1 (Apathy)	Flow_Activity1 Graphical_Activity1	0.21^∗^ (*N* = 110)	0.65^∗∗^ (*N* = 70)	0.44^∗∗^ (*N* = 104)
Activity 2 (Relaxation)	Flow_Activity2 Graphical_Activity2	0.19^∗^ (*N* = 110)	0.59^∗∗^ (*N* = 70)	0.62^∗∗^ (*N* = 104)
Activity 3 (Anxiety)	flow_Activity3 Graphical_Activity3	0.49^∗∗^ (*N* = 110)	0.60^∗∗^ (*N* = 70)	0.28^∗∗^ (*N* = 104)
Activity 4 (Flow)	Flow_Activity4 Graphical_Activity4	0.48^∗∗^ (*N* = 110)	0.71^∗∗^ (*N* = 70)	0.37^∗∗^ (*N* = 104)
Across activities	Flow_Across activities Graphical_Across activities	0.40^∗∗^ (*N* = 110)	0.49^∗∗^ (*N* = 70)	0.53^∗∗^ (*N* = 104)


*p < 0.05; **p < 0.01.

In line with our main hypothesis, correlations between flow and social identity with regards these two scales were significant and positive within each activity and across the three national samples. The results indicate that whenever the experience of flow within an activity is indicative of a relatively higher social identity, this appears for all types of self-defining activities that participants reported in the questionnaire, even though the strength of the relation slightly differs from one activity to another.

## Discussion

The present study, to our knowledge, has been the first to investigate the association between flow experience and perceived social identity, through a variety of self-defining group activities that participants freely reported based on four different combinations of skills and challenges. More specifically, the group activities reported by this sample could be categorized into some general activity category types already found in the literature (Waterman, 2004, 2005; Coatsworth et al., 2005; Sharp et al., 2007): productive and compulsory activities (e.g., study, writing a paper, teaching in school, working as an employee); social activities (e.g., talking with family, dating); structured leisure activities (i.e., sport/hobby: e.g., playing chess, music and dance, rock climbing) that have specific time, place, procedures and rules; relaxed leisure (e.g., watching TV, eating, sexual relations, window shopping); performing/fine arts/music (e.g., drama, choir, band, sculpting); and finally religious/altruistic (church attendance, volunteer work). The present paper’s results demonstrated significant positive correlations between the two main variables (flow, social identity) of interest, thus provided support for our main hypothesis in H3. To sum up, the key findings of this study are as follows.

(1) No significant effect was found either from gender or age on the subjective experience of flow. Such a result reaffirms previous descriptive findings (Csikszentmihalyi, 1990, 2000), and is in line with other research findings by various researchers (Russell, 2001; Martin and Cutler, 2002; Delle Fave and Massimini, 2005). Moreover, gender or age’s effect upon the participants’ perception of social identity was not detected; that is, the subjective experience of social identity, like flow, is found irrespective of age or gender. This finding has confirmed while also enriching Eudaimonistic identity theory (Waterman, 1990a, 1992, 1993a, 2005; Waterman et al., 2008), in that a person’s identity - whether young or older, woman or man – is enhanced through experiencing those self-defining group activities that could facilitate flow, and in further, such identity experience has been expanded further to the social level from the personal level when considering group self-defining activities.

(2) The combinations of skills and challenges within four different activity types (Activity1-*Apathy*, Activity2-*Relaxation*, Activity3-*Anxiety*, and Activity4-*Flow*) result in significantly different levels of reported flow experience, as well as the perceived social identity among activities. More specifically, the subjective flow experiences observed from the present data set confirm our hypothesis as flow is seen to be increasingly produced from *Apathy* to *Relaxation*, to *Anxiety*, and up to *Flow*. This theoretically confirms that flow, involving both challenges and skills, is best achieved when both skills and challenges are high (Csikszentmihalyi and Csikszentmihalyi, 1988; Csikszentmihalyi and LeFevre, 1989; Csikszentmihalyi, 1990). This manipulation check lends credence to both the activity classification system as well as the convergence of these categories with reported experiences of participants’ flow assessed by means of Csikszentmihalyi’s (Csikszentmihalyi, 1975, 1990) flow components. Activities participants categorize as being comprised of both high skills and high challenges are most associated with optimal experience, coherently with flow literature foundations (Csikszentmihalyi, 1975, 1990; Csikszentmihalyi and Csikszentmihalyi, 1988): flow is seen to arise when there is balance between perceived high challenges for action and high personal skills or abilities to conquer those challenges (Delle Fave et al., 2011b). Additionally, the “high skills” activities (i.e., Activity 2, Activity 4) and “high challenge” activities (i.e., Activities 3 and 4), as compared with “low skills” activities (i.e., Activity 1, Activity 3), “low challenge” activities (i.e., Activities 1 and 2), are associated with a significantly greater reported flow. Such a finding may well explain that flow encourages engaging in activities where individuals hold high skills or competences, and with high challenges for action to conquer; yet, it is worth noting that the levels of skills and challenges (no matter if low or high), are relative standings based on an individual’s personal perception. The process of activity engagement could be considered as an impetus for increasing skills or competence for achieving goals by overcoming perceived challenges, and thus it fosters both growth of skills overtime, and in turn the search for ever-increasing challenges for achieving higher goals (Nakamura and Csikszentmihalyi, 2009). From the perspective of psychological selection, the process of searching for high challenges activities for pursuing higher goals, may promote the cultivation of the individuals’ skills, interests and talents, which are considered to be platforms for personal growth (Csikszentmihalyi and Massimini, 1985). Furthermore, when experiencing high challenges activities shared with others within or across domains or cultures, an individual’s skills and competences at the societal level could be cultivated, and thusly a greater sense of psychological and social-cultural adjustment from these domains and cultures can be fostered (Massimini et al., 1988; Delle Fave et al., 2011a). Some speculations could be advanced in order to interpret why flow and social identity scores in Activity 3 (*Anxiety*) are higher than in Activity 1 and 2. This means that when challenges are higher without skills, but not the other way around, flow and social identity increase, though they do not reach the highest levels as in Activity 4 (where both skills and challenges reach their peaks). Thus, it appeared that, within a group context where social identity becomes salient (Tajfel and Turner, 1979), one of the two antecedents of flow (challenges) is more important than the other (skills). When looking at the nature of this antecedent, it is interesting to note that it is the person who had to deal with the context, the external world (namely, unbalanced challenges coming from outside). Thus, an individual seems to experience more sharing with the group (social identity) and more involvement in the socially shared activity (flow in social activities) when the outside world is challenging her/him, rather than when she/he is over-mastering it (namely, unbalanced skills). Now, in the case of socially shared activities, contrary to those conducted by a person alone, the external features and components are particularly important because the focus of the identity is precisely on the interplay of the personal, inner level with the social, group level (i.e., the outside, but also socially shared reality). It is therefore plausible that an individual experiences motivation and interest more - in the form of perceived flow and social identity- when the outside world makes some challenges that are needed for her/his own social identity. This is a theoretical speculation that needs to be tested in *ad hoc* future research.

(3) The significant positive correlations between the flow scale (Csikszentmihalyi, 1990) and the social identity measures – one comprised of questionnaire items (Mael and Ashforth, 1992) and the other of graphical multiple choice (Bergami and Bagozzi, 2000) - have confirmed a relationship between flow and social identity within each culture based on samples from three distinct countries spanning three different continents. This finding is seen to hold across the four-group self-defining activity types, and strongly supports that flow, as an optimal experience, transcends lines of culture, age, gender, and activity type (Nakamura and Csikszentmihalyi, 2002), such that group activities producing flow are associated with higher reports of social identification. Statistically speaking, the highest correlation, particularly in regards to the current scale choice and comparing among three countries is located in China, and this may be based on the fact that Chinese culture is often cited as societally collectivistic as possessing an embedded structure which promotes striving for harmony and group belonging (Hofstede, 1984; Hofstede and Bond, 1988; Earley, 1989; Huang, 2006), and thereby might be oriented to place more stake in the group level. This confirmation of the primary hypothesis provides support for extension of the implications of Eudaimonistic identity theory (Waterman, 1990b, 1992) into the social domain via psychological selection (Csikszentmihalyi and Massimini, 1985; Csikszentmihalyi and Csikszentmihalyi, 1988), where specifically those group activities infused with optimal experience are seen to be the most associated with the development of social identification on the group level. This relationship between optimal experience and the formation of group bonds is in keeping with evidence that psychological selection operates on the level beyond the individual (Delle Fave et al., 2011b), and that specifically flow experiences may provide the incentive for selection into the groups that provide the platform for such experiences. From the perspective of psychological and bio-cultural selection, the drive toward optimal experiences may motivate engagement in activities conducive to the expression of an individual’s talents and abilities, and thereby promote the selection, survival and replication of the groups that facilitate these activities. From this vantage point, elements of social identity may emerge to reinforce group cohesion and selection of bio-cultural information. These processes may hold benefit both for an individual and his/her group, community or culture at large. Finally, tests of the third hypothesis (H3) consistently show a positive significant correlation among the degree of experienced flow within group activities and the degree of perceived social identity resulting from taking part in those activities: however, there are variations among the three national samples across the four categories of group activities. Particularly, in a few of those conditions (16.7% when using OID measure and 25% when using the graphical measure of social identity), such correlations though significant are lower than in the majority of the conditions (respectively, 83.3 and 75%). For example, the US sample when in *Relaxation* activity type (and *Apathy* activity type too but only in the second of the two social identity measures), and the Spanish sample when in *Anxiety* activity type, had low values of correlation coefficients (*r* < 0.30). This was not expected and it seems to suggest that there could be some cultural differences in the intensity of the main hypothesized effect of experienced flow and perceived social identity, when the group activities diverge from the optimal balance between situational challenges and personal skills. The present data set’s results are not sufficiently clear and robust, under this specific respect, in order to elaborate more refined speculations and to allow additional possible lines of interpretation over those nuances. Future studies are thus called for in order to shed light on this particular aspect of these new results.

This study has some limitations that must be taken into account. First, the samples span differing demographic backgrounds, representative of distinct countries and cultures, yet are made up of a wide (US) or limited (China) range of occupational spread, and these features may provide both regional confounds while limiting generalizability of the findings. Second, given the correlational nature of the present study design (one-time collection without longitudinal intervals), it remains difficult to answer the question of causal relations, i.e., does a person’s perceived social identity facilitate the embedded flow experience, or is it the case that it is experiencing flow in a group activity, which in turn results in stronger social identity? The possibility remains for both cases, as optimal experience can be considered as both an antecedent and an outcome on the basis of psychological selection (Delle Fave et al., 2011b). Well-developed relationships with others, for instance, may provide “safe harbors” for the flow experience (Delle Fave et al., 2011c). These interpretations are not mutually exclusive, and the potential also remains that there are multidirectional influences such that social identity both fosters and develops through flow experiences. The current literature is yet lacking in experimental studies that might assert directionality to the observed effects that are therefore welcomed in future research.

Hence, future research may profitably explore the determination of the pathways involved in the observed relationships between flow and social identity within longitudinal scope.Still, the fact that flow and social identity are interrelated and potentially mutually supportive is an important addition for the development of flow theory toward its application at the social level. Optimal experience may both produce individual complexity, and also contribute to cultural complexity. If this is the case then flow may provide a pathway for the development of both individuation and integration in a manner that fosters positive group bonds by contributing to a balance of healthy individual agency and development in combination with societal well-being. Continued investigations would also allow for locating the associations between flow and social identity at specific group levels, i.e., gender, ethnicity, religion, nationality, political affiliation, vocations and avocations, stigmatized identities, relationships, as well as engagement within larger and non-exclusive community ties and cultural groups. When engaging in a group activity that requires personal skills, and yet in which the experience is challenging, individuals may have the opportunity to develop both themselves and the larger collective entity they are a part of. In conclusion, the findings reported here demonstrate that when flow is experienced within group self-defining activities, the perceived identity at the social level is significantly stronger than in cases where the activities are less conducive to optimal experience.

## Author Contributions

All authors listed, have made substantial, direct and intellectual contribution to the work, and approved it for publication.

## Conflict of Interest Statement

The authors declare that the research was conducted in the absence of any commercial or financial relationships that could be construed as a potential conflict of interest.

## Footnotes

1 Authors wish to acknowledge the collaboration of Mr. Mauricio Sanguinetti on this task.

2 Only in those cases in which there were not statistically significant differences observed in the preliminary analyses, were gender, age and educational level combined for subsequent manipulation check and correlational tests.
