# Supplementary material for: Optimal Experience and Optimal Identity: A Multinational Study of the Associations Between Flow and Social Identity
Source: Front Psychol. 2016 Feb 19;7:67. doi: 10.3389/fpsyg.2016.00067 (PMC4760053; doi:10.3389/fpsyg.2016.00067)
Supplement: Supplementary file 1 [file Data_Sheet_1.PDF]

We are conducting a survey about the activities you engage and we would be grateful if you could collaborate with us in filling in this questionnaire.

Please keep in mind that there are no right or wrong answers and we are interested in your own truthful opinion.

The questionnaire has only a scientific purpose and it is anonymous; your answers will be treated with total confidentiality, and only statistical aggregate data will be derived.

Please answer all the questions according to their order of presentation.

THANK YOU

FOR YOUR COLLABORATION

Section 1

1. If you wanted another person to know about **<u>who you are</u>** and **<u>what you are like as a group member</u>**, what activities you regularly engage of importance to you would you describe (minimum 5 activities maximum 10 activities)?

(1)________________________; (2)________________________;

(3)________________________; (4)________________________;

(5)________________________; (6)________________________;

(7)________________________; (8)________________________;

(9)________________________; (10)______________________;

2. From the above activities, please choose 4 (four) activities in which you regularly engage that are characterized by different combinations of skill and challenge, and write the chosen activities in each of the following boxes:

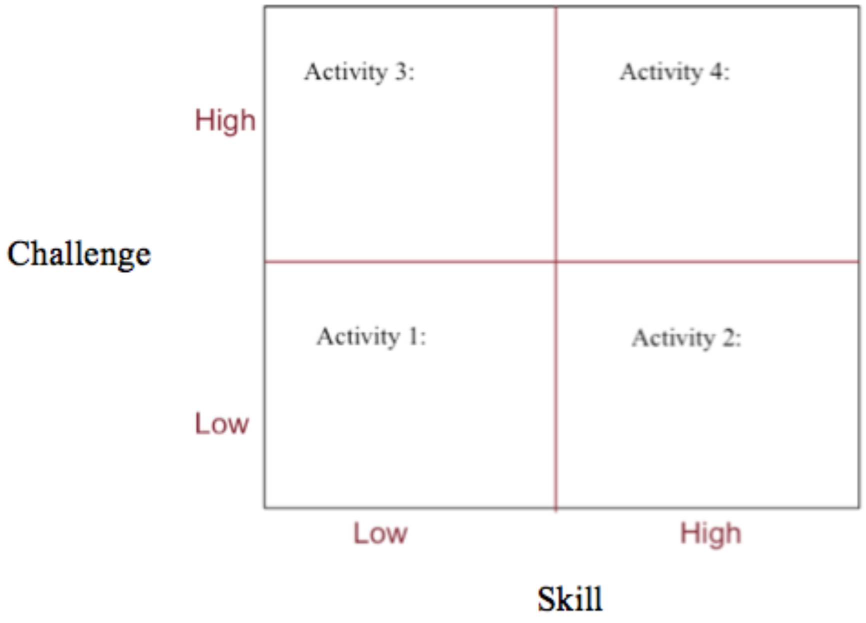

General instructions for section 2

Imagine that you are engaging in each of the above mentioned Activity 1 to 4, then focusing on each activity at time in each of the following pages, please read the items carefully, and decide how much each statement is characteristic of you when engaging in that activity by marking the score corresponding to your own degree of agreement according to the scale provided.

It is not a test, there are no right or wrong answers, and your honest and sincere answers are of great interest.

For each item in each activity, show *HOW MUCH* something is characteristic of you, by using the following scale:

**Not at all** characteristic of me	**Slightly** characteristic of me	**Moderately** characteristic of me	**Very** characteristic of me	**Completely** characteristic of me
**1**	**2**	**3**	**4**	**5**

EXAMPLE

		**Not at all** characteristic of me				**Completely** characteristic of me
		**1**	**2**	**3**	**4**	**5**
00.	I feel I have expertise in the activity.					

If you think this sentence is **slightly** characteristic of you, you should put a mark on number **2** in order to answer.

***Activity 1** (low effort & low enjoyment)*_____________*(Please write down the name of Activity 1 according to the boxes in page 2.)*

		**Not at all** characteristic of me ⟷				**Completely** characteristic of me
1.	I feel I have clear goals.	1	2	3	4	5
2.	I feel self-conscious.	1	2	3	4	5
3.	I feel in control.	1	2	3	4	5
4.	I lose track of time.	1	2	3	4	5
5.	I feel I know how well I am doing.	1	2	3	4	5
6.	I have a high level of concentration.	1	2	3	4	5
7.	I forget personal problems.	1	2	3	4	5
8.	I feel fully involved.	1	2	3	4	5
9.	When someone criticizes my group, it feels like a personal insult.	1	2	3	4	5
10.	I am very interested in what others think about my group.	1	2	3	4	5
11.	When I talk about my group, I usually say 'we' rather than 'they'.	1	2	3	4	5
12.	When someone praises my group, it feels like a personal compliment.	1	2	3	4	5
13.	My group's successes are my successes.	1	2	3	4	5
14.	If a story in the media criticized my group, I would feel embarrassed.	1	2	3	4	5

15. Imagine that one of the circles at the left in each row represents your own self-definition or identity and the other circle at the right represents group identity, please indicate which case (1,2,3,4,5,6,7,8) best describes the level of overlap between your own and the group identity (please choose only one by putting a mark on one of the 8 numbers).

1	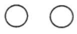	Far apart
2	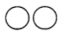	Close together but separate
3	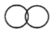	Very small overlap
4	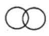	Small overlap
5	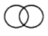	Moderate overlap
6	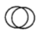	Large overlap
7	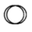	Very large overlap
8	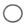	Complete overlap

Activity 2 (high effort & low enjoyment)____________(Please write down the name of Activity 2 according to the boxes in page 2.)

		**Not at all** characteristic of me ⟷			**Completely** characteristic of me
1. I feel I have clear goals.	1	2	3	4	5
2. I feel self-conscious.	1	2	3	4	5
3. I feel in control.	1	2	3	4	5
4. I lose track of time.	1	2	3	4	5
5. I feel I know how well I am doing.	1	2	3	4	5
6. I have a high level of concentration.	1	2	3	4	5
7. I forget personal problems.	1	2	3	4	5
8. I feel fully involved.	1	2	3	4	5
9. When someone criticizes my group, it feels like a personal insult.	1	2	3	4	5
10. I am very interested in what others think about my group.	1	2	3	4	5
11. When I talk about my group, I usually say 'we' rather than 'they'.	1	2	3	4	5
12. When someone praises my group, it feels like a personal compliment.	1	2	3	4	5
13. My group's successes are my successes.	1	2	3	4	5
14. If a story in the media criticized my group, I would feel embarrassed.	1	2	3	4	5

15. Imagine that one of the circles at the left in each row represents your own self-definition or identity and the other circle at the right represents group identity, please indicate which case (1,2,3,4,5,6,7,8) best describes the level of overlap between your own and the group identity (please choose only one by putting a mark on one of the 8 numbers).

1	○ ○	Far apart
2	○○	Close together but separate
3	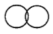	Very small overlap
4	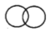	Small overlap
5	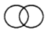	Moderate overlap
6	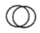	Large overlap
7	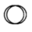	Very large overlap
8	○	Complete overlap

Activity 3 *(low effort & high enjoyment)____________(Please write down the name of Activity 3 according to the boxes in page 2.)*

		**Not at all** characteristic of me ←→				**Completely** characteristic of me
1.	I feel I have clear goals.	1	2	3	4	5
2.	I feel self-conscious.	1	2	3	4	5
3.	I feel in control.	1	2	3	4	5
4.	I lose track of time.	1	2	3	4	5
5.	I feel I know how well I am doing.	1	2	3	4	5
6.	I have a high level of concentration.	1	2	3	4	5
7.	I forget personal problems.	1	2	3	4	5
8.	I feel fully involved.	1	2	3	4	5
9.	When someone criticizes my group, it feels like a personal insult.	1	2	3	4	5
10.	I am very interested in what others think about my group.	1	2	3	4	5
11.	When I talk about my group, I usually say 'we' rather than 'they'.	1	2	3	4	5
12.	When someone praises my group, it feels like a personal compliment.	1	2	3	4	5
13.	My group's successes are my successes.	1	2	3	4	5
14.	If a story in the media criticized my group, I would feel embarrassed.	1	2	3	4	5

15. Imagine that one of the circles at the left in each row represents your own self-definition or identity and the other circle at the right represents group identity, please indicate which case (1,2,3,4,5,6,7,8) best describes the level of overlap between your own and the group identity (please choose only one by putting a mark on one of the 8 numbers).

1	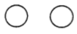	Far apart
2	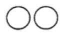	Close together but separate
3	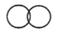	Very small overlap
4	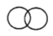	Small overlap
5	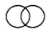	Moderate overlap
6	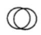	Large overlap
7	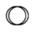	Very large overlap
8	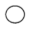	Complete overlap

Activity 4 (*high effort & high enjoyment)*____________*(Please write down the name of Activity 4 according to the boxes in page 2.)*

		**Not at all** characteristic of me ⟷			**Completely** characteristic of me
1. I feel I have clear goals.	1	2	3	4	5
2. I feel self-conscious.	1	2	3	4	5
3. I feel in control.	1	2	3	4	5
4. I lose track of time.	1	2	3	4	5
5. I feel I know how well I am doing.	1	2	3	4	5
6. I have a high level of concentration.	1	2	3	4	5
7. I forget personal problems.	1	2	3	4	5
8. I feel fully involved.	1	2	3	4	5
9. When someone criticizes my group, it feels like a personal insult.	1	2	3	4	5
10. I am very interested in what others think about my group.	1	2	3	4	5
11. When I talk about my group, I usually say 'we' rather than 'they'.	1	2	3	4	5
12. When someone praises my group, it feels like a personal compliment.	1	2	3	4	5
13. My group's successes are my successes.	1	2	3	4	5
14. If a story in the media criticized my group, I would feel embarrassed.	1	2	3	4	5

15. Imagine that one of the circles at the left in each row represents your own self-definition or identity and the other circle at the right represents group identity, please indicate which case (1,2,3,4,5,6,7,8) best describes the level of overlap between your own and the group identity (please choose only one by putting a mark on one of the 8 numbers).

1	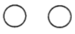	Far apart
2	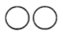	Close together but separate
3	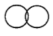	Very small overlap
4	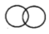	Small overlap
5	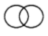	Moderate overlap
6	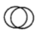	Large overlap
7	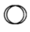	Very large overlap
8	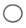	Complete overlap

Section 3

1. Age: ____________________________________

2. Gender: male ( ) female ( )

3. What is your current marital status?
   single ( ) married ( ) divorced ( ) living with another ( ) other _____________

4. What is your citizenship? ____________________________________

5. Where do you live now? ____________________________________

6. Which of the following best describes the area you live in?
   urban ( ) sub-urban ( ) rural ( )

7. What is your highest level of education you have completed?
   High school ( ) Bachelor's degree( ) Master's degree ( ) Doctoral degree ( )

8. What is your occupation? ____________________________________

9. How would you rate your English level?
   very poor ( ) poor ( ) intermediate ( ) good ( ) excellent ( )

10. What is your first language? ____________________________________

THANK YOU SO MUCH!
